# Supplementary material for: The transcription factor NF-Y participates to stem cell fate decision and regeneration in adult skeletal muscle
Source: Nat Commun. 2021 Oct 14;12:6013. doi: 10.1038/s41467-021-26293-w (PMC8516959; doi:10.1038/s41467-021-26293-w)
Supplement: Supplementary file 1 — Supplementary Information [file 41467_2021_26293_MOESM1_ESM.pdf]

The transcription factor NF-Y participates to stem cell fate decision and regeneration in adult skeletal muscle

Supplementary Information

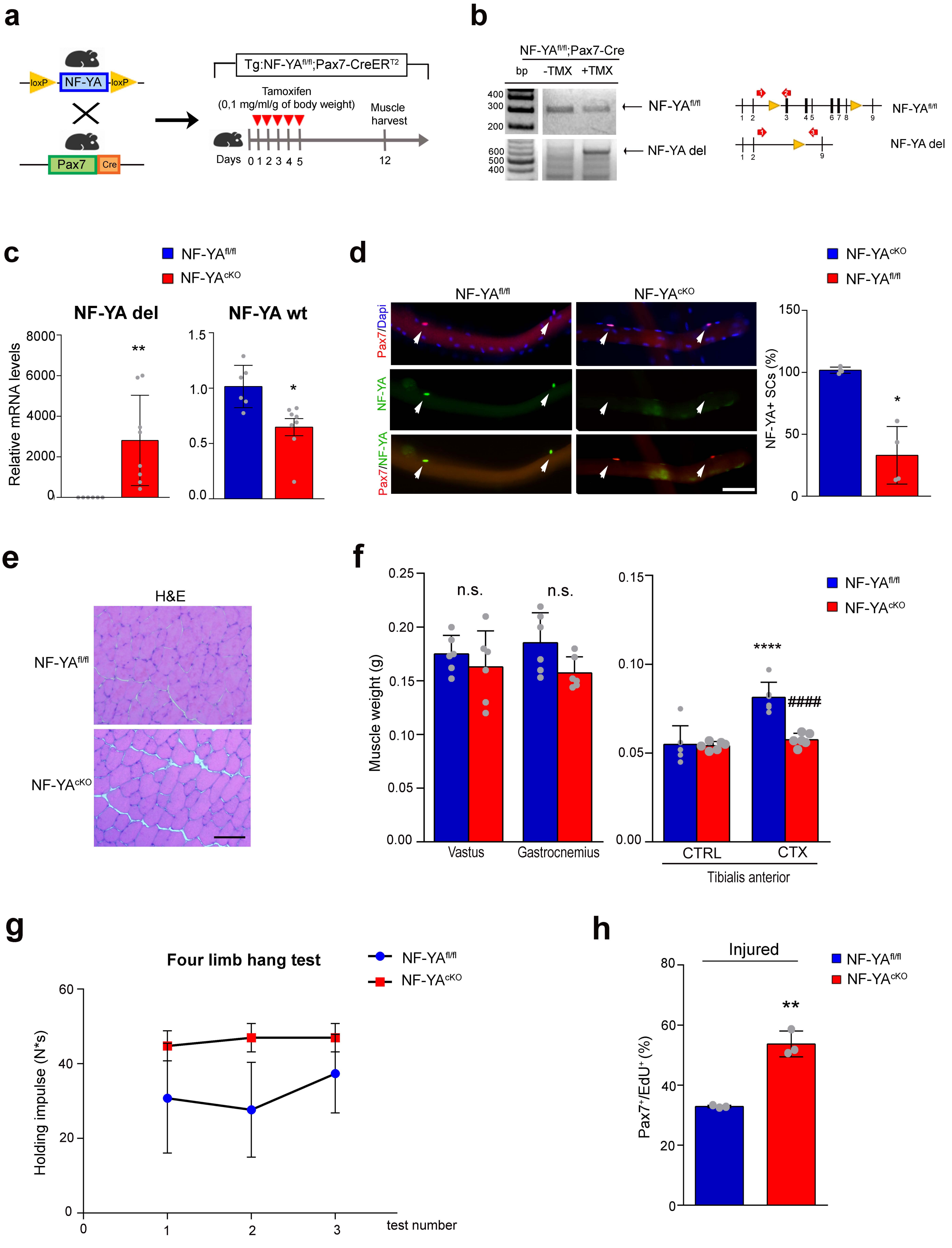

Supplementary Figure 1

Supplementary Fig.1: Characterization of NF-YA^{cKO} mouse model. (**a**) Schematic representation of mice crossed to obtain NF-YA$^{fl/fl}$;Pax7-CreER^{T2} F1 generation and Tamoxifen administration to induce NF-YA deletion in NF-YA^{cKO} mice. (**b**) Left panel: PCR analysis of genomic DNA from NF-YA$^{fl/fl}$;Pax7Cre Tamoxifen-treated and untreated mice. The arrows indicate NF-YA$^{fl/fl}$ amplicon, generated by primers 1 and 2, and NF-YA deleted (del) amplicon, generated by primers 1 and 3. PCR analysis was tested for each animal used for subsequent analyses. Right panel: Schematic representation of NF-YA coding gene, loxP elements (yellow triangles) and primers used for PCR analysis (red arrows). (**c**) qRT-PCR analysis of NF-YA deleted (del) (p=0.0100) and wt (p=0.0066) transcripts in SCs isolated from NF-YA^{cKO} *versus* NF-YA$^{fl/fl}$ mice, arbitrarily set at 1. Data represent mean ± s.d. (two-tailed unpaired t-test:*p<0.05, **p< 0.01; n=3 mice). (**d**) Left panel: Representative immunofluorescence images of NF-YA and Pax7 in EDL myofibers at day 0 (d0) following isolation from NF-YA$^{fl/fl}$ and NFYA^{cKO} mice. Right panel: Quantification of NF-YA+ SCs identified by Pax7 positivity in NF-YA^{cKO} *versus* NF-YA$^{fl/fl}$ myofibers, arbitrarily set at 100%. Data represent mean ± s.d. (two-tailed unpaired t-test: p=0.011; *p<0.05; n=4 mice). (**e**) Representative images of NF-YA^{cKO} and NF-YA$^{fl/fl}$ TA cross-sections stained with H&E. Scale bar: 50 µm. (**f**) Left panel: weight of vastus and gastrocnemius uninjured muscles of NF-YA^{cKO} and NF-YA$^{fl/fl}$ mice. Right panel: weight of tibialis anterior muscle after 60 days from CTX injury of NF-YA^{cKO} and NF-YA$^{fl/fl}$ mice. Data represent means ± s.d. (two-tailed unpaired t-test: *p<0.05, **p< 0.01; n=6 mice). (**g**) Four limb grip test performed on adult NF-YA^{cKO} and NF-YA$^{fl/fl}$ mice 60 days after CTX injury. Three independent tests were performed. Data represent means ± s.d. (two-tailed unpaired t-test; n.s.= not significant. n=5 mice). (**h**) Percentage of EdU+/Pax7+ cells in TA muscle sections following 5 days from CTX-injury. Data represent mean ± s.d. (two-tailed unpaired t-test: **p<0.01; n=3 mice).

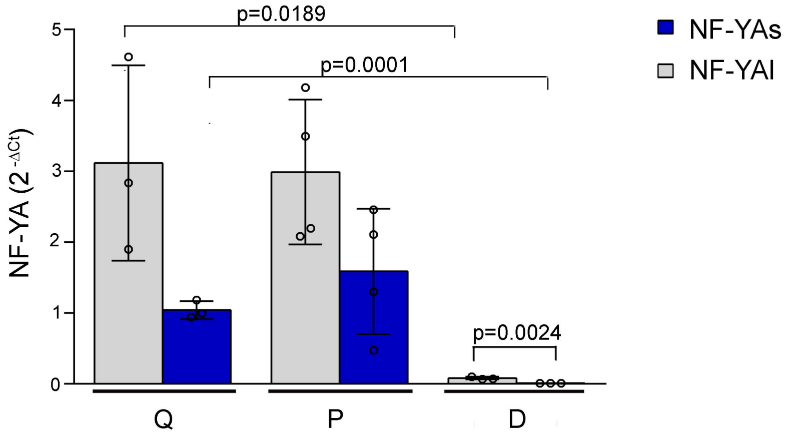

Supplementary Figure 2

Supplementary Fig. 2: Transcriptional analysis of enriched SC-cultures*.* mRNA levels of NF-YA isoforms (NF-YAl and NF-YAs) in Pax3/GFP+ sorted SCs quiescent (Q), proliferating (P) and differentiating (D). Data represent mean ± s.d. (two-tailed unpaired t-test: p values are shown in figure; n=3 mice).

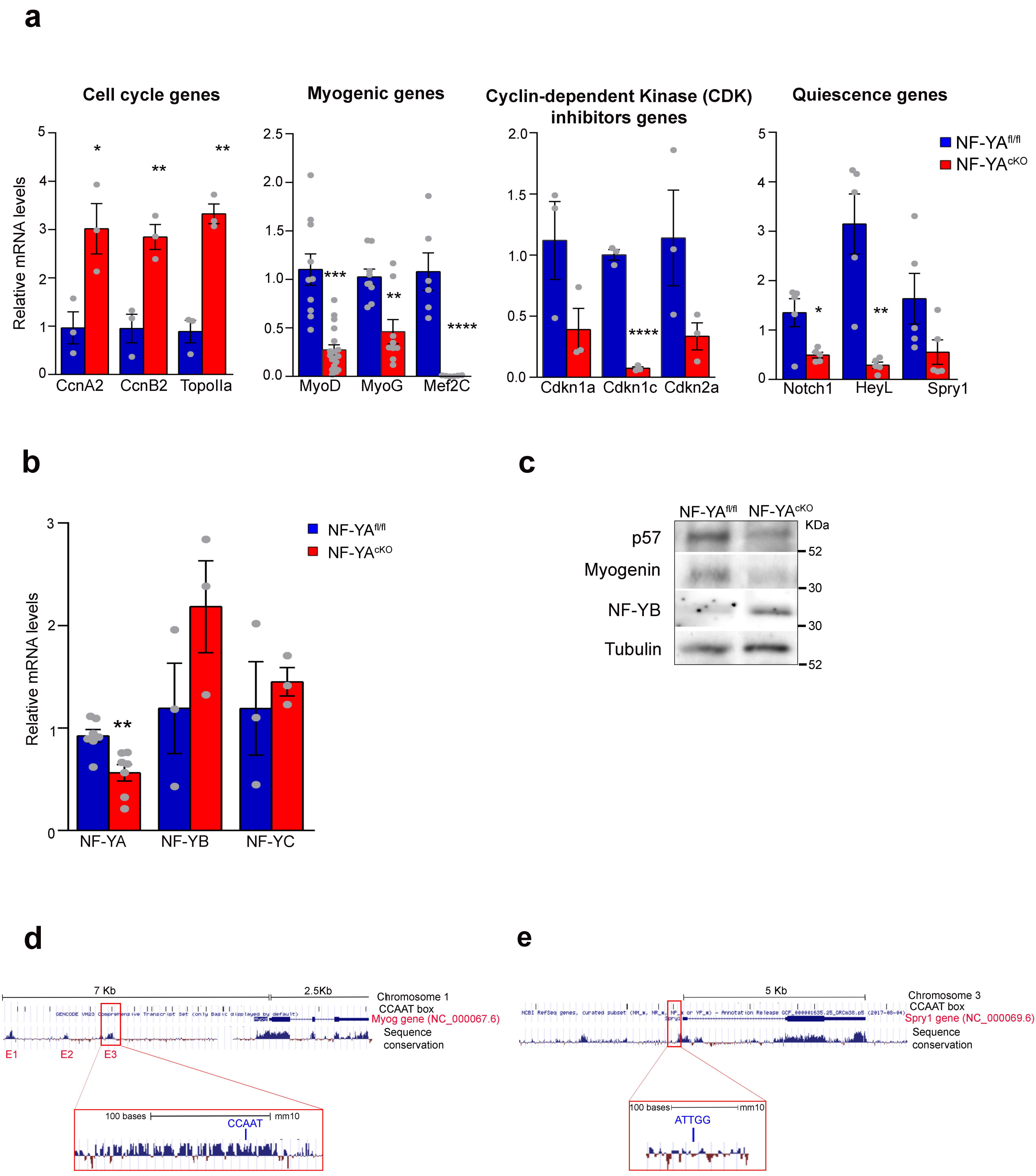

Supplementary Figure 3

Supplementary Fig. 3: Validation of transcriptional modulation of direct target genes in NF-YA^{cKO} mouse. (**a**) qRT-PCR analysis of the indicated genes in NF-YA$^{fl/fl}$ and NF-YA^{cKO} SCs. Data represent mean ± s.d. (two-tailed unpaired t-test: *CcnA2* p=0.0290, *CcnB2* p=0.0085, *TopoIIa* p=0.0014, *MyoD* p=0.0003, *MyoG* p=0.0016, *Mef2C* p<0.0001, *Cdkn1c* p<0.0001, *Notch* p=0.0179, *HeyL* p=0.0016; *p<0.05, **p<0.01, ***p<0.001, ****p<0.0001, n=5 mice). (**b**) Relative mRNA levels measured by qRT-PCR of NF-YA, NF-YB and NF-YC genes in NF-YA$^{fl/fl}$ and NF-YA^{cKO} SCs. Data represent mean ± s.d. (two-tailed unpaired t-test: *NF-YA* p=0.0041;**p<0.05, n=5 mice). (**c**) Western blot of whole extracts from SCs isolated from NF-YA$^{fl/fl}$ and NF-YA^{cKO} mice with anti-p57, anti-Myogenin and anti-NF-YB antibodies. Tubulin expression was used as loading control. n= 3 experiments. (**d**) Schematic representation (UCSC Genome browser) of *Myogenin* gene and upstream enhancer elements (E1, E2, E3). The enlargement shows the NF-Y binding element (CCAAT) within the E3 region. (**e**) Schematic representation (UCSC Genome browser) of *Sprouty1* gene and upstream promoter region. The enlargement shows the NF-Y binding element (CCAAT) at about 200 bp upstream of TSS.

Supplementary Tables

Supplementary Table 1: Sequence of each primer used for mice genotyping.

Genomic qPCR	*FORWARD*	*REVERSE*
NF-YA$^{fl/fl}$	GTAAGTCAGGCTCCAGGG	AGGCAAGGCAGATTTAGGAAGGTC
NF-YA^{del}	GTAAGTCAGGCTCCAGGG	GGTTGTCAGGATGTTCGCAG
Pax7-Cre^{ERT2}	CCACACCTCCCCCTGAACCTGAAACATAAA	GAATTCCCCGGGGAGTCGCATCCTGCGG

Supplementary Table 2: Sequence of each primer used in real time qPCR for mRNA analysis.

RT-qPCR	*FORWARD*	*REVERSE*
Rps15	ACCTACAAACCCGTGAAGCA	CCAGGGACCAAAACCAGTC
NF-YA wt	ACGAAGGAAATACCTCCATGAGTC	CTTCCCCACGCTTCCGT
NF-YA deleted	CAGACCCTCCAGGTAGATCCAA	TGTGGTTAGGAAACTCGGATGA
Pax7	GCTACCAGTACAGCCAGTATG	GTCACTAAGCATGGGTAGATG
eMyHC	TTGATGCCAAAACCTACTGCT	GGGTCCTGCTGTCTTCTGTC
Myf5	CACCACCAACCCTAACCAGA	GTTCTCCACCTGTTCCCTCA
Myogenin	GAGCCCCACTTCTATGATGG	GTCCCCAGTCCCTTTTCTTC
Mef2c	AGTACACCGAGTACAACGAGC	GCCTGTGTTACCTGCACTTGG
Ccnb2	TCTCTGATGCTCTGCTCTGC	CCGAAACTTGGAATGGACTT
Ccna2	CAGAACTCATTCGGCTCTCA	GCCAAGGGAAAAGGAAGAAG
Topo-IIα	ACGGAATGACAAGCGAGAAG	AAACAACAACCGAGCCAAAG
Cdkn1a	CAAGGAGACCCCAAAGTCC	GAGGCAGATTTCTATCACTCC
Cdkn1c	TCCACCTCCATCCACTGC	AGAACCGCTGGGACTTAAC
Cdkn2a	CGCTTTTGTTCGGTTTTGTT	TCCAGGGGCTTATGATTCTG
NF-YA	ACAGTATCACCGCATCCTTAAGA	CCTTCGTTCCTTTGGGATTT
NF-YB	AAGCGGAAGACAATCAATGG	ATCTGTGGCGGAGACTGCT
NF-YC	GCTACCAATGCCCAACAGAT	CCCTCAGTCTCCAGTCACCT
Mre11a	CTTATCCGACTACGGGTGGA	TTTCCCTTTTGTTCCCTGTG
Brca1	GAAACACGCCAAATGTCTGA	GATACGCTGGTGCTCTCCTC
Rad51	GGTTAGAGCAGTGTGGCATAA	TAGTTCCTTCTTCGGTGCATAAG
Notch1	GCAACTGTCCTCTGCCATATAC	GTCTTCAGACTCCTTGCATACC
HeyL	CATCACTCCCTGAAGACGAAAG	GGAAGGGTTGTAGCCTTAGATG
Spry1	CACACTTCGCTAGTGGTGATT	TGGTCTAGGGACAGAATCGTAG
Birc5	CCGAGAACGAGCCTGATTT	GAGTGCTTTCTATGCTCCTCTATC
Bcl2l2	GTGGGTAGAAGCTTTGGTAGTT	GCTGGATAGAGAGACCCTAGAA

Supplementary Table 3: Sequence of each primer used in real time qPCR for ChIP analysis.

RT-qPCR	***FORWARD***	***REVERSE***
Chrm7	ATAAAGGCTTGGCACTCGTC	CAGTTCCCTTTGCTTGATCC
Ccnb1	CAGGCATAGAGCCTGACCTC	GTCTGCCGGGCTTAGGTTTA
Ccnb2	AAATACAAGCCAGCCAATCAA	GACGAGGCACAGCCACTC
Topo-IIα	CCTTCCTCATTGGTCAGATTTT	GACTCGCTCTCATTGGCTCT
Cdkn1c promoter	CCAAGCTGGACAGGACAAG	AAGCGTTCCATCGCTGTT
Cdkn1c 5'UTR	GATCTGACCTCAGACCCAATTC	TGCTCAGAGACCTGCTCA
Myogenin enhancer	CAGGTCAGAGCTGATGGAT	CACTAGCTGCCCTCTGATG
Spry1	AAACTCAACTCTAAGGGTGGCT	GAGGAGCGGGCATTCCA
