# Supplementary material for: The transcription factor NF-Y participates to stem cell fate decision and regeneration in adult skeletal muscle
Source: Nat Commun. 2021 Oct 14;12:6013. doi: 10.1038/s41467-021-26293-w (PMC8516959; doi:10.1038/s41467-021-26293-w)
Supplement: Supplementary file 3 — Reporting Summary [file 41467_2021_26293_MOESM3_ESM.pdf]

Corresponding author(s): Carol Imbriano

Last updated by author(s): Aug 31, 2021

Reporting Summary

Nature Research wishes to improve the reproducibility of the work that we publish. This form provides structure for consistency and transparency in reporting. For further information on Nature Research policies, see our Editorial Policies and the Editorial Policy Checklist.

Statistics

For all statistical analyses, confirm that the following items are present in the figure legend, table legend, main text, or Methods section.

n/a	Confirmed	
☐	☒	The exact sample size (*n*) for each experimental group/condition, given as a discrete number and unit of measurement
☒	☐	A statement on whether measurements were taken from distinct samples or whether the same sample was measured repeatedly
☐	☒	The statistical test(s) used AND whether they are one- or two-sided<br>*Only common tests should be described solely by name; describe more complex techniques in the Methods section.*
☒	☐	A description of all covariates tested
☒	☐	A description of any assumptions or corrections, such as tests of normality and adjustment for multiple comparisons
☐	☒	A full description of the statistical parameters including central tendency (e.g. means) or other basic estimates (e.g. regression coefficient) AND variation (e.g. standard deviation) or associated estimates of uncertainty (e.g. confidence intervals)
☐	☒	For null hypothesis testing, the test statistic (e.g. *F*, *t*, *r*) with confidence intervals, effect sizes, degrees of freedom and *P* value noted<br>*Give P values as exact values whenever suitable.*
☒	☐	For Bayesian analysis, information on the choice of priors and Markov chain Monte Carlo settings
☒	☐	For hierarchical and complex designs, identification of the appropriate level for tests and full reporting of outcomes
☒	☐	Estimates of effect sizes (e.g. Cohen's *d*, Pearson's *r*), indicating how they were calculated

Our web collection on statistics for biologists contains articles on many of the points above.

Software and code

Policy information about availability of computer code

Data collection

- Images were acquired using a Zeiss AxioSkop 40 fluorescence microscope and EVOS M5000 (Thermo Fisher Scientific)
-qRT-PCR was conducted using Biorad CFX connect thermocycler.
-RNA Sequencing was performed using Illumina NextSeq high-output cartridge (2X75).
-Western blot detection and acquisition were performed by Amersham Imager AI680 RGB (GE Healthcare)

Data analysis

- G-power software for preliminary calculation of the number of animals needed for the study.
- Beckman Coulter EXPO32 software for cell cycle analysis.
-ImageJ v1.52a software for cells and centrally nucleated fibers counting, CSA and infiltrated area measurements.
-GraphPad Prism 6.0 was used for graph generation and all molecular data analyses.
- BioRender2021 (Biorender.com) and Adobe Photoshop CC2019 (ver.20.0.1 20181029.r.41 2018/010/29) were used to create graphic experimental scheme.
-RNA-seq data was aligned to mouse transcriptome (mm10) using bowtie2 within RSEM v1.3.1 and quantified inplace (command: rsem-calculate-expression -p $NPROC --paired-end --bowtie2 --estimate-rspd --no-bam-output --append-names ${name}_R{1,2}_001.fastq.gz $REF $RESULTS/$onlyname).
- DESeq2 v1.38 R package was employed to normalize read counts and to compute differentially expressed genes.

```
#create a DESeq model with the experiment values and the experimental design
DDS<-DESeqDataSetFromMatrix(colData=condition,countData=data,design=~condition)
#DESeq model extimation
DDS<-DESeq(DDS)
#compute differential expressed genes
res<-results(DDS, contrast=c("condition","KO","WT"))
```

Over-represented TFs motifs in the promoter of up- and down-regulated genes were estimated with Pscan web tool (Ver. 1.5 - 19 January

2018)(http://159.149.160.88/pscan/), employing non redundant JASPAR 2018 set of matrices and selecting -450/+50 bp near TSS.
Enriched GO Terms and Pathways, in the two gene list, were computed by KOBAS 3.0 run by command line (run_kobas.py --infile=$entrez_list --intype=id:ncbigene --species=mmu) set for Fisher exact Test and Benjamini and Hochberg p-value correction (both default).
R environment employed was version 3.6.3.

For manuscripts utilizing custom algorithms or software that are central to the research but not yet described in published literature, software must be made available to editors and reviewers. We strongly encourage code deposition in a community repository (e.g. GitHub). See the Nature Research guidelines for submitting code & software for further information.

Data

Policy information about availability of data

All manuscripts must include a data availability statement. This statement should provide the following information, where applicable:

- Accession codes, unique identifiers, or web links for publicly available datasets
- A list of figures that have associated raw data
- A description of any restrictions on data availability

RNA-seq data that support the findings of this study is deposited in Gene Expression Omnibus (GEO) database with the accession code GSE154017. GEO record is currently public.
Fig. 1a,1b,1c,1e, 1g, 1h, 1i, 2b,2c, 2d, 3b, 3c, 3d, 4a,4 b, 4c, 4d, 4f, 4g, 5c, 5d, 5e, 5i, 5h, 6c,6d, 6e, 6f, 7c, 7d, 7e, 7f, 7g, Supplementary Fig. 1b, 1c, 1d, 1f, 1g, 1h, 2, 3a, 3b, 3c have associated raw data in the Source Data file. All other data supporting the findings of this study are available upon request.

Field-specific reporting

Please select the one below that is the best fit for your research. If you are not sure, read the appropriate sections before making your selection.

[x] Life sciences [ ] Behavioural & social sciences [ ] Ecological, evolutionary & environmental sciences

For a reference copy of the document with all sections, see nature.com/documents/nr-reporting-summary-flat.pdf

Life sciences study design

All studies must disclose on these points even when the disclosure is negative.

Sample size	Sample size for animal studies was precalculated by G-power software (power 80% and alpha-error 0.05). In particular, for Cardiotoxin experiments, sample size was precalculated by two-way Anova F-test (power 80% and alpha-error 0.05, effect size 0.5 and degree of freedom 1).
Data exclusions	For molecular analysis, we used pre-exclusion criteria based on the efficiency of NF-YA deletion induced by Tamoxifen administration. By means of PCR analysis of NF-YA deleted transcript and genotype, samples that did not show significant levels of NF-YA deletion were excluded for subsequent analysis.
Replication	The same procedures were performed at least three times independently and data were collected for statistical analysis. All the replicates were successful.
Randomization	Mice were allocated into experimental groups on the basis of their genotype. Within each group, mice were randomly chosen. For all experiments, we used the same age and gender of control and KO mice from the same litter whenever possible. For cell experiments, we randomly counted multiple fields or cells per group for quantification.
Blinding	For immunofluorescence/histological data collection , we performed the experiments in a blinded way randomly counting multiple fields per group and calculated the number of positively stained cells per field. For molecular analysis (qRT-PCR and Western Blot) blinding was not applicable.

Reporting for specific materials, systems and methods

We require information from authors about some types of materials, experimental systems and methods used in many studies. Here, indicate whether each material, system or method listed is relevant to your study. If you are not sure if a list item applies to your research, read the appropriate section before selecting a response.

Materials & experimental systems

n/a	Involved in the study
☐	☒ Antibodies
☒	☐ Eukaryotic cell lines
☒	☐ Palaeontology and archaeology
☐	☒ Animals and other organisms
☒	☐ Human research participants
☒	☐ Clinical data
☒	☐ Dual use research of concern

Methods

n/a	Involved in the study
☒	☐ ChIP-seq
☐	☒ Flow cytometry
☒	☐ MRI-based neuroimaging

Antibodies

Antibodies used

Rabbit anti-NF-YA (C15310261, Diagenode, mouse anti-NF-YA (G2) (sc-17753, Santa Cruz Biotechnology), rabbit anti-NF-YA (1:100, Thermo Fisher Scientific), rabbit anti-NF-YA (H-209), (1:100, sc-10779 Santa Cruz Biotechnology), mouse anti-Pax7 (Developmental Studies Hybridoma Bank), mouse anti-MyHCII (MF20) (Developmental Studies Hybridoma Bank), rabbit anti-Myogenin (M-225) (sc-576 Santa Cruz Biotechnology), mouse anti-eMyHC (Developmental Studies Hybridoma Bank), goat anti-Actin (I-19) (sc-1616, Santa Cruz Biotechnology), mouse anti-Vinculin (V4504, Sigma Aldrich), rabbit anti-GAPDH (sc-47727, Santa Cruz Biotechnology), mouse anti-Tubulin (6031 ,Proteintech Europe), mouse anti-p57Kip2 (66794, Proteintech Europe), rabbit anti-NF-YB (GeneSpin), rabbit anti-laminin (ab11575, Abcam), rabbit anti-MyoD (C-20) (sc-304 ,Santa Cruz Biotechnology), rabbit anti-phospho(S10)-histone H3 (06-750 Millipore), goat anti-histone H3 (sc-8654 Santa Cruz Biotechnology), rabbit anti-MRE11A (E-AB-11403 Elabscience), rabbit anti-RAD51 (H-92) (sc-8349 Santa Cruz Biotechnology), rabbit anti-phospho(S139)-H2AX (2577 Cell Signaling), rabbit anti-histone H2A (Ab18255 Abcam)rabbit anti-MyoD (M-318) (sc-760 Santa Cruz Biotechnology), rabbit anti-MyoD (8943-1-AP Proteintech), mouse anti-Myogenin (M3559 Dako), rabbit anti-cleaved Caspase3 (Asp175) (9661 Cell Sinaling), anti-rabbit AlexaFluor 488 (A21206, Life Technologies), anti-mouse AlexaFluor 568 (A10037, Life Technologies), goat anti-mouse (A90116P, Bethyl Laboratories), goat anti-rabbit (G21234, ThermoFisher Scientific), donkey anti-goat (A50101P, Bethyl Laboratories), rabbit IgG (sc-2027, Santa Cruz Biotechnology).

Validation

All antibodies used are commercially available and validated for mouse species and for the applications tested in this study by the manufacturers. The validation information for application and antigen specificity being provided in the respective data sheets from the manufacturers or on the manufacturers' websites.
NF-YB antibody (GeneSpin) has been previously validated by collaborators and by our group (Dolfini D, Zambelli F, Pedrazzoli M, Mantovani R, Pavesi G. A high definition look at the NF-Y regulome reveals genome-wide associations with selected transcription factors. Nucleic Acids Res. 2016;44(10):4684-4702. doi:10.1093/nar/gkw096; Benatti P, Basile V, Dolfini D, Belluti S, Tomei M, Imbriano C. NF-Y loss triggers p53 stabilization and apoptosis in HPV18-positive cells by affecting E6 transcription. Oncotarget. 2016;7 (29):45901-45915. doi:10.18632/oncotarget.9974).

Animals and other organisms

Policy information about studies involving animals; ARRIVE guidelines recommended for reporting animal research

Laboratory animals

Mus musculus, C57B6, males, 6-8 weeks old were used for experiments. All animals were housed at controlled temperature and humidity on a 12 h light-dark cycle and provided water and food ad libitum. The Pax7-CreERT2 mice were kindly provided to G.M. by Prof. Shahragim Tajbakhsh, Institut Pasteur (Paris, France). NF-YAfl/fl mice were a gift from Nobuyuki Nukina's laboratory at Juntendo University Graduate School of Medicine (Tokyo, Japan). The NF-YAfl/fl mice were bred with the Pax7-CreERT2 mice to generate NF-YAfl/fl;Pax7-Cre ERT2 mice.

Wild animals

This study did not involve wild animals.

Field-collected samples

This study did not involve samples collected from the field.

Ethics oversight

The experiments involving mice were approved by National Institute of Health (Ministero della Salute) (n. 404/2015-PR and 699/2019-PR) and by the Institutional Animal Care Committee.

Note that full information on the approval of the study protocol must also be provided in the manuscript.

Flow Cytometry

Plots

Confirm that:

☐ The axis labels state the marker and fluorochrome used (e.g. CD4-FITC).

☐ The axis scales are clearly visible. Include numbers along axes only for bottom left plot of group (a 'group' is an analysis of identical markers).

☐ All plots are contour plots with outliers or pseudocolor plots.

☒ A numerical value for number of cells or percentage (with statistics) is provided.

Methodology

Sample preparation	For satellite cells sorting, muscles were dissected, mechanically cut and enzymatically digested at 37°C with a solution of Collagenase I (100 mg/ml, Sigma-Aldrich), Dispase (500 mg/ml,Gibco), and DNaseI (100 mg/ml, Roche) in PBS (Sigma-Aldrich). Undigested tissue was precipitated for 5 min, the supernatant centrifuged for 5 min at 1200 g and cell pellet resuspended and blocked with 10% donkey serum (Sigma-Aldrich) in PBS for 15 min at room temperature (RT).<br>Cells were incubated 30 min on ice with primary antibody detecting SM/C-2.6 antigen, biotinylated, 1:200 . After extensive washes, cells were incubated with Streptavidin-APC (BD Pharmingen, 1:500) and CD45-FITC (Rat Anti-Mouse, 30-F11, BD Pharmingen,1:100), 20 min on ice. The cells were washed again and sorted with a FACS Aria cell sorter (Beckman).<br><br>Flow cytometric cell cycle analysis of isolated satellite cells was performed after collecting cells by centrifugation, washed with PBS and resuspended in 400µL of PI solution (0.1%, Triton, 3.4M Na citrate, 50µg/mL Propidium Iodide). After 30 min of incubation, cells were analyzed for DNA content using cytofluorimeter.
Instrument	Satellite cells sorting: FACS Aria III cell sorter (BD Biosciences)<br>Cell cycle analysis: Coulter Epics XL MCL Beckman Coulter
Software	Satellite cells sorting: BD FacsDIVA software<br>Cell cycle analysis: Beckman Coulter EXPO32 software
Cell population abundance	The abundance of SM/C-2.6 positive and CD45 negative cells (Satellite Cells) is about 0,75% of the total cell population in the WT mice
Gating strategy	Satellite cells sorting: satellites cells sorting is based on positivity for SM/C-2.6 antigen and negativity for CD45.<br>Cell cycle analysis: while running the cytometer, Forward Scatter (FS) and Side Scatter (SS) were set to identify single live cells and exclude debris. For analysis, the first gate on the single cell population was applied to the scatter plot to gate out obvious debris. Then, combined gates were applied to the PI histogram plot. Quantification of the cell percentage in each cell cycle phase was performed by using markers set within the analysis program.

☐ Tick this box to confirm that a figure exemplifying the gating strategy is provided in the Supplementary Information.
